# Chemical Bath Deposition of ZnO/ZnGa_2_O_4_ Core–Shell Nanowire Heterostructures Using Partial Chemical Conversion

**DOI:** 10.3390/nano14120991

**Published:** 2024-06-07

**Authors:** Guislain Hector, Estelle Appert, Hervé Roussel, Anna Bujak, Eirini Sarigiannidou, Vincent Consonni

**Affiliations:** Université Grenoble Alpes, CNRS, Grenoble INP, LMGP, F-38000 Grenoble, France; guislain.hector@grenoble-inp.fr (G.H.); estelle.appert@grenoble-inp.fr (E.A.); herve.roussel@grenoble-inp.fr (H.R.); anna.bujak@grenoble-inp.fr (A.B.); eirini.sarigiannidou@grenoble-inp.fr (E.S.)

**Keywords:** ZnO nanowires, ZnGa_2_O_4_, chemical bath deposition, core–shell heterostructure

## Abstract

The development of innovative heterostructures made of ZnO nanowires is of great interest for enhancing the performances of many devices in the fields of optoelectronics, photovoltaics, and energy harvesting. We report an original fabrication process to form ZnO/ZnGa_2_O_4_ core–shell nanowire heterostructures in the framework of the wet chemistry techniques. The process involves the partial chemical conversion of ZnO nanowires grown via chemical bath deposition into ZnO/ZnGa_2_O_4_ core–shell nanowire heterostructures with a high interface quality following their immersion in an aqueous solution containing gallium nitrate heated at a low temperature. The double-step process describing the partial chemical conversion relies on successive dissolution and reaction mechanisms. The present finding offers the possibility to fabricate ZnO/ZnGa_2_O_4_ core–shell nanowire heterostructures at low temperatures and over a wide variety of substrates with a large surface area, which is attractive for nanostructured solar cells, deep-UV photodetectors, and piezoelectric devices.

## 1. Introduction

As a compound semiconductor with attractive properties, zinc gallate, also known as zinc gallium oxide (ZnGa_2_O_4_), has received increasing interest in recent years. Its wide-band gap energy (4.4–5.0 eV), its relatively high electron mobility (up to 100 cm^2^·V^−1^·s^−1^), and its high stability make it an interesting candidate for deep UV solar-blind photodetectors [1]. ZnGa_2_O_4_ crystallizes into a cubic spinel structure at ambient conditions, in which the divalent Zn cations occupy 1/8 of the tetrahedral sites, while the trivalent Ga cations occupy 1/2 of the octahedral sites in the normal spinel crystal [1]. ZnGa_2_O_4_ further shows an n-type electrical conductivity, but recent reports have demonstrated its potential p-type conductivity, opening the way for the development of p–n homojunction-based devices [2].

ZnGa_2_O_4_ nanoparticles and thin films have typically been grown using a large number of methods, including pulsed-laser deposition, magnetron sputtering, metal–organic chemical vapor deposition, thermal synthesis, sol–gel process, and hydrothermal synthesis [3,4,5,6,7,8]. Among them, hydrothermal synthesis offers several advantages, as a low-cost, low-temperature, and surface-scalable method. A first classical approach has consisted of mixing zinc and gallium salts in an aqueous solution in order to induce the precipitation of ZnGa_2_O_4_ [8]. A second more original approach has involved the chemical conversion of a solid phase serving as a sacrificial material. This method could involve zinc nanoparticles, but gallium-based compounds have been much more explored [9]. Indeed, the standard Gibbs energy of reaction is favorable to form the ZnGa_2_O_4_ phase instead of the mixture of the ZnO and Ga_2_O_3_ phases [10]. Accordingly, several reports have shown the chemical conversion of gallium-based compounds to ZnGa_2_O_4_ [11,12,13,14]. Chen et al. reported the synthesis of ZnGa_2_O_4_ with cubic or octahedral shapes from GaOOH and Ga_2_O_3_, following the addition of Zn(NO_3_)_2_ under basic media. It was noticed that a better crystallinity of the particles originated from the GaOOH powder. However, an incomplete chemical conversion was revealed for this reaction operating at a lower pH (pH = 8) [11]. Later, Yan et al. reported the chemical conversion of GaOOH to ZnGa_2_O_4_ with a cubic shape, following the addition of Zn(CH_3_COO)_2_. The pH of the solution was not adjusted and was assumed to be in the acidic range [12]. Liang et al. reported the chemical conversion of GaOOH and α-Ga_2_O_3_ nanowires grown on FTO by adding Zn(CH_3_COO)_2_ [13]. The GaOOH nanowires were shown to be converted into ZnGa_2_O_4_ nanowires with a cubic shape, in a similar manner to Ref. [12]. However, the chemical conversion of α-Ga_2_O_3_ nanowires led to the formation of ZnGa_2_O_4_ nanotubes through the Kirkendall effect, which, in turn, kept the 1D morphology. A similar result was demonstrated by Lu et al., when partially converting GaN nanowires into GaN/ZnGa_2_O_4_ core–shell nanowire heterostructures [14]. The chemical conversion was performed with the help of Zn(NO_3_)_2_ as a zinc source and HMTA as a pH buffer. Interestingly, the reaction occurred at a lower temperature (85 °C) compared to the other methods described in Refs. [11,12,13], for which a higher temperature (>150 °C) was required. Until now, the chemical conversion of gallium-based compounds is therefore the only known method to obtain ZnGa_2_O_4_ with a 1D morphology on a substrate using the hydrothermal synthesis method. However, the formation of vertically aligned dense arrays of gallium-based nanowires using a low-cost, low-temperature, and surface-scalable method is a tricky task. Only a couple of substrates are known to induce this type of growth, which is required to form nanowires [15,16]. On the other hand, the formation of vertically aligned dense arrays of ZnO nanowires has been widely explored and demonstrated on a wide variety of substrates [17]. However, the chemical conversion of ZnO to ZnGa_2_O_4_ and its related mechanism have not been reported so far.

In this article, we present the partial chemical conversion of ZnO nanowires to ZnO/ZnGa_2_O_4_ core–shell nanowire heterostructures by using a low-temperature hydrothermal synthesis method. The influence of the gallium precursor concentration and of the initial pH (pH_0_) of the solution on the ZnGa_2_O_4_ shell growth is carefully investigated. We show the good coverage of the ZnGa_2_O_4_ shell up to the complete embedment of ZnO nanowires by the ZnGa_2_O_4_ shell. Then, the crystallinity of the ZnO/ZnGa_2_O_4_ core–shell nanowire heterostructures is optimized using different thermal annealing processes. The complete crystallization of the ZnGa_2_O_4_ shell is shown to proceed at 400 °C under air at ambient pressure. Eventually, the mechanisms responsible for the partial chemical conversion of ZnO nanowires into ZnO/ZnGa_2_O_4_ core–shell nanowire heterostructures are discussed.

## 2. Materials and Methods

### 2.1. Deposition Techniques

**Figure 1:**
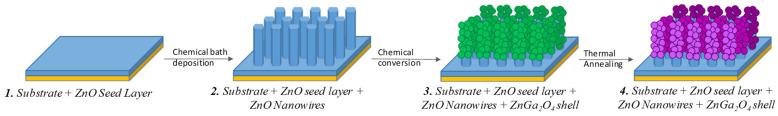
Schematic of the fabrication process of the ZnO/ZnGa_2_O_4_ core–shell nanowire heterostructures using wet chemistry.

The fabrication process of the ZnO/ZnGa_2_O_4_ core–shell nanowire heterostructures is presented in Figure 1. ZnO nanowire arrays were prepared using a double-step process of a sol–gel process and a chemical bath deposition. The ZnO seed layer was first deposited on a silicon substrate by dip coating, using the conditions described in Ref. [18], including an equimolar concentration of 375 mM for zinc acetate dihydrate [Zn(Ac)_2_·2H_2_O, Sigma Aldrich (St. Louis, MO, USA)] and monoethanolamine [MEA, Sigma Aldrich], as well as a withdrawal speed of 3.3 mm/s. Then, the silicon substrate coated with the ZnO seed layer was placed face-down in a sealed reactor to form ZnO nanowire arrays via chemical bath deposition using the conditions described in Ref. [19], including an equimolar concentration of 30 mM for zinc nitrate hexahydrate [Zn(NO_3_)_2_·6H_2_O, Sigma-Aldrich] and hexamethylenetetramine [HMTA, Sigma-Aldrich]. ZnO/ZnGa_2_O_4_ core–shell nanowire heterostructures were obtained by immersing the ZnO nanowire arrays in a sealed reactor containing a dissolved gallium nitrate [Ga(NO_3_)_3_·xH_2_O, Sigma Aldrich] solution, with x considered to be 8 at a concentration varying in the range of 16–100 mM. The pH_0_ of the solution was adjusted to a range of values varying from 8.56 to 9.65 by the addition of NaOH (Sigma Aldrich). The sealed reactor was kept at 90 °C in a regular oven for 3 h.

### 2.2. Annealing Process

The ZnO/ZnGa_2_O_4_ core–shell nanowire heterostructures were eventually treated thermally under air at ambient pressure using an annealing temperature of 400 °C, 600 °C, and 900 °C. The temperature ramp was carried out in successive steps every 100 °C, from 200 °C to the annealing temperature, with a rising temperature ramp set at 10 °C/min. The dwell time was set to 30 min. At the end of the annealing process, the samples were cooled down under air to room temperature.

### 2.3. Characterization Techniques

The morphological properties of ZnO nanowires and ZnO/ZnGa_2_O_4_ core–shell nanowire heterostructures were investigated using field-emission scanning electron microscopy (FESEM) images in top-view and cross-sectional view configurations using a ZEISS GeminiSEM 300 instrument (Carl Zeiss, Oberkochen, Germany). X-ray diffraction (XRD) patterns were collected with a BRUKER D8 Advance diffractometer using Cu K_α1_ radiation according to the Bragg–Brentano configuration. Cross-sectional transmission electron microscopy (TEM) lamella were prepared using the semi-automated polishing tripod technique with the MultiPrepTM system (Allied High Tech Products, Inc., Cerritos, CA, USA). A GATAN PIPS II system was used for the final polishing. High-resolution TEM (HRTEM) images were recorded with a JEOL JEM 2010 LaB_6_ microscope (Tokyo, Japan) operating at 200 kV, with a 0.19 nm point-to-point resolution.

## 3. Results

### 3.1. Effect of the Gallium Nitrate Concentration of the Solution on the ZnGa_2_O_4_ Shell Formation

**Figure 2:**
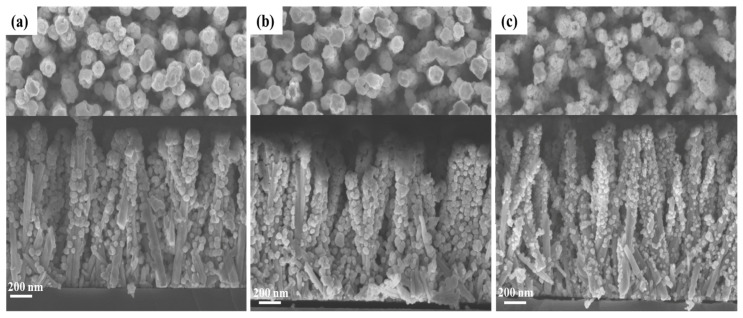
Top-view and cross-sectional view FESEM images of ZnO nanowire arrays coated with the ZnGa_2_O_4_ shell after a partial chemical conversion using a solution with a Ga(NO_3_)_3_ concentration of (**a**) 16 mM, (**b**) 50 mM, and (**c**) 100 mM for a given pH_0_ value of 9.27.

The effect of the Ga(NO_3_)_3_ concentration of the solution on the morphological properties of the ZnGa_2_O_4_ shell and its uniformity was investigated using FESEM and XRD measurements. The pH_0_ of the solution was set to 9.27 at room temperature in order to (i) avoid the dissolution of ZnO nanowires during the addition of the Ga(NO_3_)_3_ solution with a pH_0_ of ≈2.5 [17], (ii) favor the predominant formation of Ga(OH)_4_^−^ anions acting as reacting species [20], and (iii) keep the positive surface charge of the top face and sidewalls of ZnO nanowires made of the polar *c*-plane and non-polar *m*-planes, respectively, with a point-of-zero-charge of 8.75 and 10.2 at room temperature [21,22]. The present conditions are designed to establish attractive electrostatic interactions between the positively charged surfaces of ZnO nanowires and the Ga(OH)_4_^−^ reacting species [13,14]. Typical FESEM images of ZnO nanowires coated with the ZnGa_2_O_4_ shell obtained for Ga(NO_3_)_3_ concentrations of 16, 50, and 100 mM are presented in Figure 2. The FESEM images show the formation of spherical-shaped nanoparticles (i.e., nanospheres) around the ZnO nanowires, regardless of the Ga(NO_3_)_3_ concentration. For each Ga(NO_3_)_3_ concentration, the nanospheres exhibit a mean size lying in the range of 30–50 nm. The nanospheres preferentially form on the top of ZnO nanowires, but their formation is also seen on the sidewalls of ZnO nanowires. For a Ga(NO_3_)_3_ concentration of 16 and 50 mM, the nanospheres coalesce to form a fairly continuous shell around the ZnO nanowires. Instead, for a Ga(NO_3_)_3_ concentration of 100 mM, the nanospheres do not form a continuous shell. The shell composed of nanospheres extends from the top of the ZnO nanowires to their bottom, close to the ZnO seed layer, regardless of the Ga(NO_3_)_3_ concentration. However, the density of nanospheres is lower and lower as the position gets closer to the bottom of the ZnO nanowires, regardless of the Ga(NO_3_)_3_ concentration. The base of ZnO nanowires thus remains relatively bare. The effect is particularly pronounced when using the Ga(NO_3_)_3_ concentration of 16 mM. Also, it is worth noting that the top of some ZnO nanowires has been dug during the chemical conversion process, possibly indicating a partial dissolution process from the top polar *c*-planes.

**Figure 3:**
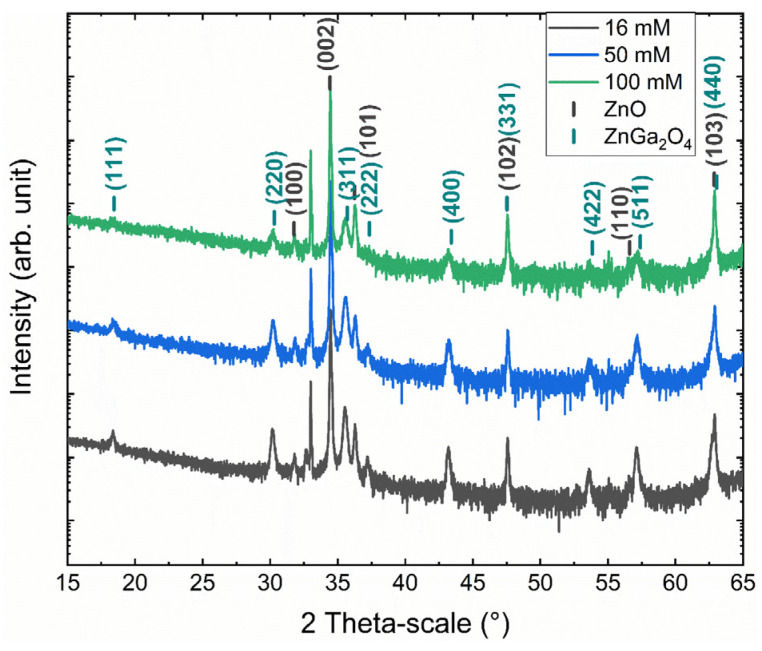
XRD patterns of ZnO nanowire arrays coated with the ZnGa_2_O_4_ shell after a chemical conversion using a solution with a Ga(NO_3_)_3_ concentration of 16 mM, 50 mM, and 100 mM for a given pH_0_ value of 9.27.

The XRD measurements of ZnO nanowires coated with the ZnGa_2_O_4_ shell obtained for Ga(NO_3_)_3_ concentrations of 16, 50, and 100 mM are presented in Figure 3. The XRD patterns show the presence of ZnO nanowires with the wurtzite structure (i.e., the P63mc space group) through the diffraction peaks at 31.77°, 34.42°, 36.25°, 47.54°, 56.60°, and 62.86° corresponding to the (100), (002), (101), (102), (110), and (103) planes, respectively, according to the 00-036-1451 ICDD file. The remaining diffraction peaks at 18.44°, 30.31°, 35.70°, 37.34°, 43.41°, 47.51°, 53.85°, 57.40°, and 63.04° are all attributed to the ZnGa_2_O_4_ shell with the cubic spinel structure (i.e., the Fd-3m space group) according to the 00-038-1240 ICDD file and correspond to the (111), (220), (311), (222), (400), (331), (422), (511), and (440) planes, respectively. Interestingly, the XRD analysis does not reveal any residual traces of the GaOOH and Ga(OH)_3_ phases, which could have been obtained during the chemical conversion.

**Figure 4:**
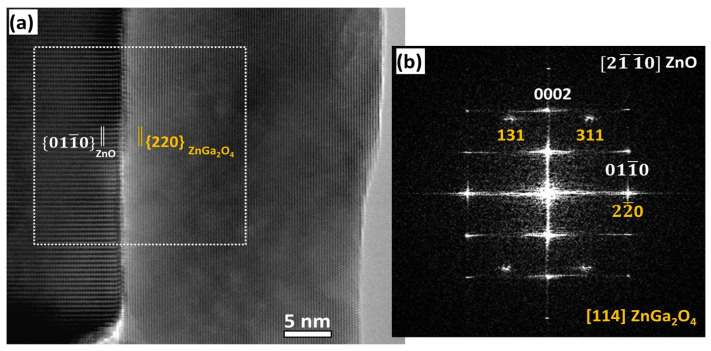
(**a**) HRTEM image at the interface of a ZnO/ZnGa_2_O_4_ core–shell nanowire heterostructure projected along the [2-1-10] ZnO // [114] ZnGa_2_O_4_ zone axes. (**b**) Corresponding FFT image of the interfacial area. The {01-10} planes of the ZnO core are aligned with the {220} planes of the ZnGa_2_O_4_ shell. The TEM analysis is illustrated with a ZnO nanowire coated with the ZnGa_2_O_4_ shell after a partial chemical conversion using a solution with a Ga(NO_3_)_3_ concentration of 50 mM for a given pH_0_ value of 9.27.

The cubic spinel structure of the ZnGa_2_O_4_ shell was also shown in HRTEM images, as depicted in Figure 4. A typical HRTEM image of a ZnO/ZnGa_2_O_4_ core–shell nanowire heterostructure projected along the [2-1-10] ZnO // [114] ZnGa_2_O_4_ zone axes is presented in Figure 4a, where the alignment of the {01-10} planes of the ZnO core with the {220} planes of the ZnGa_2_O_4_ shell is clearly revealed. The interface between the ZnO core and the ZnGa_2_O_4_ shell is quite abrupt, indicating that the partial chemical conversion is a powerful method to form ZnO/ZnGa_2_O_4_ core–shell nanowire heterostructures with a high quality. The Fast Fourier Transform (FFT) image taken at the interfacial area, as presented in Figure 4b, further confirms the aforementioned observations.

The influence of the Ga(NO_3_)_3_ concentration of the solution on the formation of the ZnGa_2_O_4_ shell by the partial chemical conversion of ZnO nanowires in aqueous solution eventually reveals that a value of 50 mM is considered to be the optimal Ga(NO_3_)_3_ concentration to provide the best trade-off between the nanosphere size, density, and coverage.

### 3.2. Effect of the pH_0_ of the Solution on the ZnGa_2_O_4_ Shell Formation

**Figure 5:**
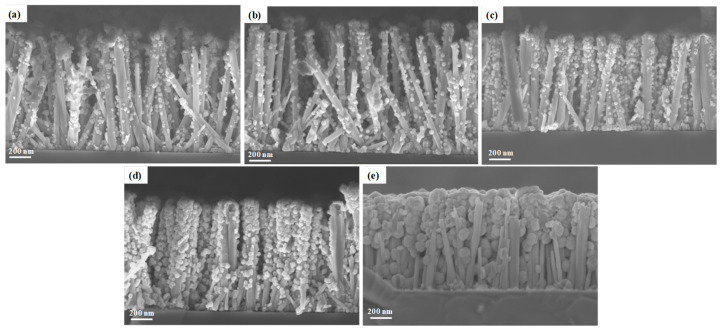
Top-view and cross-sectional view FESEM images of ZnO nanowire arrays coated with the ZnGa_2_O_4_ shell after a partial chemical conversion using a solution with a pH_0_ value of (**a**) 8.56, (**b**) 8.64, (**c**) 9.00, (**d**) 9.27, and (**e**) 9.65 for a given Ga(NO_3_)_3_ concentration of 50 mM.

The effect of the pH_0_ of the solution in a narrow range favoring attractive electrostatic interactions between the positively charged surfaces of ZnO nanowires and the Ga(OH)_4_^−^ reacting species on the morphological properties of the ZnGa_2_O_4_ shell and its uniformity was investigated using FESEM and XRD measurements. The Ga(NO_3_)_3_ concentration of the solution was set to 50 mM. Typical FESEM images of ZnO nanowires coated with the ZnGa_2_O_4_ shell obtained for pH_0_ values of 8.56, 8.64, 9.00, 9.27, and 9.65 are presented in Figure 5. The FESEM images again show the formation of nanospheres around the ZnO nanowires, regardless of the pH_0_ value. For pH_0_ values of 8.56, 8.64, 9.00, and 9.27, the size of the nanospheres lies in the range of 20–50 nm. Interestingly, the mean size of nanospheres progressively increases as the pH_0_ value is increased from 8.56 to 9.27. In contrast, for the pH_0_ value of 9.65, the size of the nanospheres is much larger and jumps to the range of 90–130 nm. Overall, the nanospheres are distributed homogeneously around the ZnO nanowires. Again, they preferentially form on the top of the ZnO nanowires, but their formation is also seen on the sidewalls of the ZnO nanowires. For pH_0_ values of 8.56 and 8.64, the nanospheres only coalesce on the top of the ZnO nanowires to form a continuous shell. In contrast, isolated nanospheres sparsely occur on the sidewalls of the ZnO nanowires, with no contact between them. The density of nanospheres around the ZnO nanowires significantly increases for a pH_0_ value of 9.00, such that coalescence starts proceeding. For a pH_0_ value of 9.27, the nanospheres coalesce to form a fairly continuous shell around the ZnO nanowires. At this pH_0_, the shell covers up to 2/3 of the ZnO nanowire height, but their base still remains relatively bare. For a pH_0_ value of 9.65, the nanospheres completely impregnate the ZnO nanowire arrays from the bottom to their top and hence the morphology of the core–shell nanowire heterostructure is lost at the benefit of a full 2D encapsulation acting as a planarization process.

**Figure 6:**
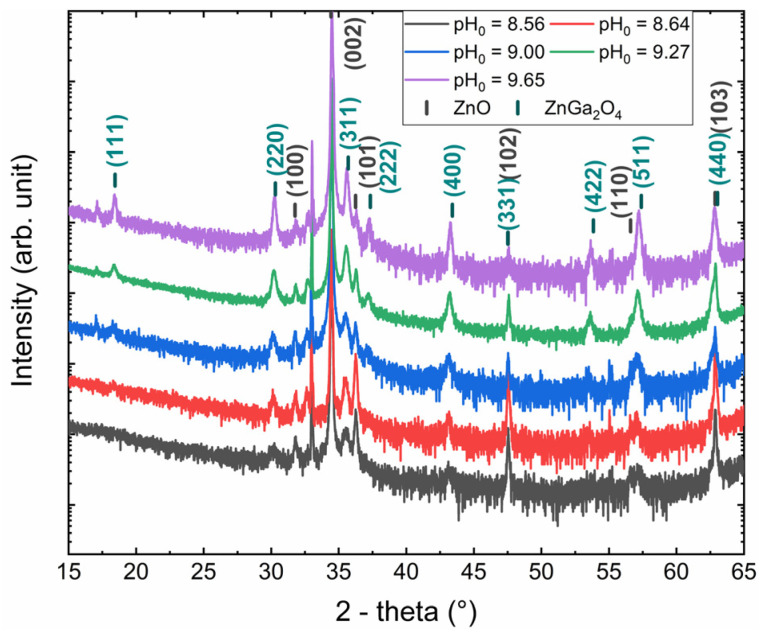
XRD patterns of ZnO nanowire arrays coated with the ZnGa_2_O_4_ shell after a chemical conversion using a solution with a pH_0_ value of 8.56, 8.64, 9.00, 9.27, and 9.65 for a given Ga(NO_3_)_3_ concentration of 50 mM.

The XRD measurements of the ZnO nanowires coated with the ZnGa_2_O_4_ shell obtained for pH_0_ values of 8.56, 8.64, 9.00, 9.27, and 9.65 are presented in Figure 6. The presence of ZnO nanowires with the wurtzite structure is revealed through the diffraction peaks at 31.77°, 34.42°, 36.25°, 47.54°, 56.60°, and 62.86° corresponding to the (100), (002), (101), (102), (110), and (103) planes, respectively. The remaining diffraction peaks at 18.44°, 30.31°, 35.70°, 37.34°, 43.41°, 47.51°, 53.85°, 57.40°, and 63.04° are, respectively, assigned to the (111), (220), (311), (222), (400), (331), (422), (511), and (440) planes of the ZnGa_2_O_4_ shell with the cubic spinel structure. Interestingly, the intensity of the diffraction peaks related to the ZnGa_2_O_4_ shell strongly increases as the pH_0_ value is increased from 8.56 to 9.65, which is in agreement with the fact that the nanospheres have an increasing size. Again, no residual traces of the GaOOH and Ga(OH)_3_ phases are detected through the XRD analysis.

The influence of the pH_0_ of the solution on the formation of the ZnGa_2_O_4_ shell by the partial chemical conversion of ZnO nanowires in aqueous solution finally shows that a value of 9.27 is considered as the optimal pH_0_ to lead to the best compromise between nanosphere size, density, and coverage.

### 3.3. Study of the Crystallization Process of the ZnGa_2_O_4_ Shell

**Figure 7:**
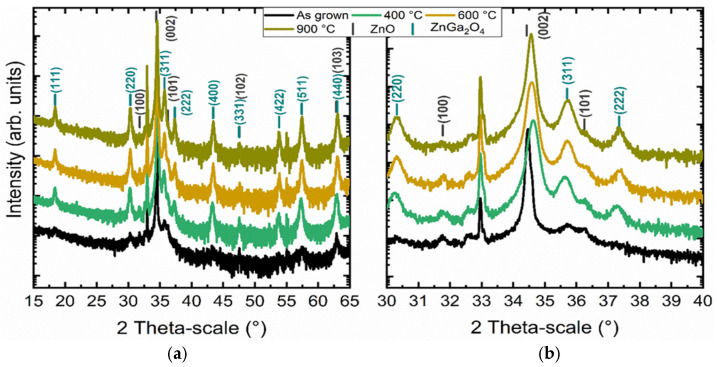
(**a**) XRD patterns of ZnO nanowires coated with the ZnGa_2_O_4_ shell before and after thermal annealing under air at 400 °C, 600 °C, and 900 °C and (**b**) magnification between 30° and 40°. The chemical conversion parameters were set to a Ga(NO_3_)_3_ concentration of 50 mM and a pH_0_ value of 9.27.

The study of the formation of a ZnGa_2_O_4_ shell by the partial chemical conversion of ZnO nanowires in aqueous solution allowed for the creation of a ZnGa_2_O_4_ shell. Although a crystallized ZnGa_2_O_4_ phase was identified using XRD, the presence of amorphous or poorly crystallized regions in the shell cannot be excluded. It is thus specifically important to study the evolution of the crystallinity of the ZnGa_2_O_4_ phase after a post-deposition thermal annealing process under air at ambient pressure. The annealing time was kept below 10 h to limit the development of the insulating character of ZnGa_2_O_4_ [23]. The structural characterization of the annealed ZnO/ZnGa_2_O_4_ core–shell nanowire heterostructures was carried out using XRD measurements. The XRD patterns collected before and after thermal annealing at 400 °C, 600 °C, and 900 °C are presented in Figure 7. The diffraction peaks at 31.77°, 34.42°, 36.25°, 47.54°, and 62.86° again correspond to the (100), (002), (101), (102), and (103) planes of the ZnO nanowires. The diffraction peak at 32.96° is attributed to the harmonic of the (002) plane coming from the Si substrate. The remaining diffraction peaks at 18.44°, 30.31°, 35.70°, 37.34°, 43.41°, 47.51°, 53.85°, 57.40°, and 63.04° are, respectively, assigned to the (111), (220), (311), (222), (400), (331), (422), (511), and (440) planes of the ZnGa_2_O_4_ shell. The XRD analysis does not highlight any residual traces from other phases. The intensity of the diffraction peaks related to the ZnGa_2_O_4_ shell before thermal annealing is particularly low, revealing its low crystallinity. Conversely, the intensity of the diffraction peaks related to the ZnGa_2_O_4_ shell strongly increases after thermal annealing, showing a higher crystallinity. However, the diffraction peaks of the ZnGa_2_O_4_ shell roughly keep the same intensity, regardless of the annealing temperature. Therefore, a well-crystallized ZnGa_2_O_4_ shell can be obtained from the annealing temperature of 400 °C under air, with the annealing time beginning at about 2 h. It is further worth noting the broadening of the diffraction peak at 34.42°, corresponding to the (002) plane of the ZnO nanowires, which may result from the creation of defects at their top, hence revealing their progressive dissolution during the immersion process.

## 4. Discussion

**Figure 8:**
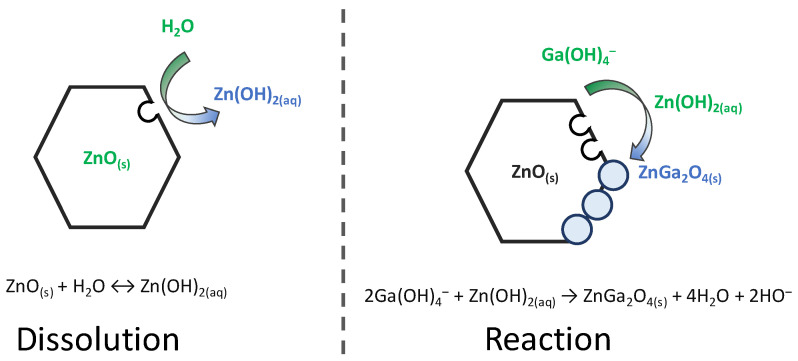
Schematic diagram of the double-step process describing the partial chemical conversion of ZnO into ZnGa_2_O_4_ through successive dissolution and reaction mechanisms.

The chemical bath deposition of ZnO/ZnGa_2_O_4_ core–shell nanowire heterostructures using a partial chemical conversion of ZnO nanowires in aqueous solution has been investigated using FESEM, HRTEM, and XRD measurements. By analogy with the chemical conversion of GaN, α-GaOOH, and α-Ga_2_O_3_ in aqueous solution to ZnGa_2_O_4_ [13,14], a schematic diagram of the double-step process is summarized in Figure 8. The partial chemical conversion of ZnO nanowires relies on the successive dissolution and reaction mechanisms, which are highly dependent upon the pH value. Under neutral and moderately basic conditions, the progressive dissolution of the surfaces of ZnO nanowires operates to release Zn(OH)_2(aq)_ species in aqueous solution [24]. These Zn(II) species are assumed to react with the predominant Ga(III) species—Ga(OH)_4_^−^ ion complexes [25]. This results in the formation of the ZnGa_2_O_4_ shell as nanospheres, for which the shape depends on the dissolution process of the surfaces of the ZnO nanowires, while the size depends on their reactivity. It is well known that the polar *c*-planes located on the top of the ZnO nanowires exhibit a greater chemical reactivity than the non-polar *m*-planes on their sidewalls [26]. As a result, the ZnGa_2_O_4_ shell composed of nanospheres is more dense and thicker on the top of the ZnO nanowires. This particularity has already been used to form ZnO/TiO_2_ epitaxial core–shell nanowire heterostructures with a varying thickness [27], as well as ZnO/Zn_2_TiO_4_ core–shell nanowire heterostructures, and TiO_2_ nanotubes [28]. In the present case, the fabrication of ZnO/ZnGa_2_O_4_ core–shell nanowire heterostructures opens perspectives for nanostructured solar cells [29], deep-UV photodetectors [30], and piezoelectric devices [31], for which the management of charge carriers and interface properties is of primary importance. In the case of deep-UV photodetectors, the present ZnO/ZnGa_2_O_4_ core–shell nanowire heterostructures may benefit from the absorption of deep-UV with the ZnGa_2_O_4_ shell and from the electron transport with the ZnO nanowires [1,30]. In the case of piezoelectric devices, the ZnGa_2_O_4_ shell may passivate the surface states of ZnO nanowires, affecting the density of surface traps and hence the piezoelectric potential [1,31].

## 5. Conclusions

In summary, the development of innovative core–shell heterostructures, along with the control of the interfacial properties of ZnO nanowires, are of great interest for the enhancement of the performances of many devices. We have reported an original fabrication process to form ZnO/ZnGa_2_O_4_ core–shell nanowire heterostructures in the framework of the wet chemistry technique. The process involves the partial chemical conversion of ZnO nanowires grown via chemical bath deposition into ZnO/ZnGa_2_O_4_ core–shell nanowire heterostructures following their immersion in an aqueous solution containing Ga(NO_3_)_3_ heated at 90 °C. The ZnGa_2_O_4_ shell has been found to form uniformly, following a coalescence process of isolated nanospheres. It exhibits a pure spinel structure and a high interface quality with ZnO nanowires. The crystallinity of the ZnGa_2_O_4_ shell has been improved by a post-deposition thermal annealing at 400 °C under air at ambient pressure. The effect of the pH_0_ value and Ga(NO_3_)_3_ concentration of the solution on the structural properties of the ZnGa_2_O_4_ shell has further been revealed, showing the possibility to tune its morphology and thickness. The double-step process describing the partial chemical conversion has been discussed, relying on the progressive dissolution of the surfaces of the ZnO nanowires releasing Zn(OH)_2(aq)_ species and their reaction with the predominant Ga(OH)_4_^−^ ion complexes. The present findings offer the possibility to fabricate ZnO/ZnGa_2_O_4_ core–shell nanowire heterostructures at low temperatures and over a wide variety of substrates with a large surface area, which is attractive for nanostructured solar cells, deep-UV photodetectors, and piezoelectric devices.

## Data Availability

The data that support the findings of this study are available from the corresponding authors upon reasonable request.
